# Loss of Function of E-Cadherin in Embryonic Stem Cells and the Relevance to Models of Tumorigenesis

**DOI:** 10.1155/2011/352616

**Published:** 2010-12-09

**Authors:** Lisa Mohamet, Kate Hawkins, Christopher M. Ward

**Affiliations:** Core Technology Facility, Faculty of Medical and Human Sciences, The University of Manchester, 46 Grafton Street, Manchester M13 9NT, UK

## Abstract

E-cadherin is the primary cell adhesion molecule within the epithelium, and loss of this protein is associated with a more aggressive tumour phenotype and poorer patient prognosis in many cancers. Loss of E-cadherin is a defining characteristic of epithelial-mesenchymal transition (EMT), a process associated with tumour cell metastasis. We have previously demonstrated an EMT event during embryonic stem (ES) cell differentiation, and that loss of E-cadherin in these cells results in altered growth factor response and changes in cell surface localisation of promigratory molecules. We discuss the implication of loss of E-cadherin in ES cells within the context of cancer stem cells and current models of tumorigenesis. We propose that aberrant E-cadherin expression is a critical contributing factor to neoplasia and the early stages of tumorigenesis in the absence of EMT by altering growth factor response of the cells, resulting in increased proliferation, decreased apoptosis, and acquisition of a stem cell-like phenotype.

## 1. E-Cadherin Protein Structure and Function

**Figure 1:**
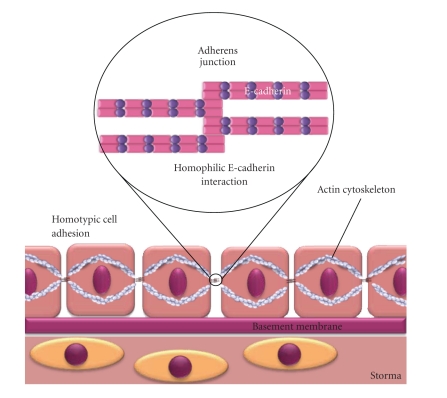
*Diagrammatic representation of homophilic E-cadherin interaction and homotypic cell adhesion within the epithelium.* E-cadherin cis-dimers form transhomodimers with E-cadherin molecules on neighbouring cells to facilitate epithelial integrity. Note that the exact mechanism of homophilic E-cadherin interaction is unclear. For clarity, only E-cadherin is represented within adherens junctions.

Cadherins are a family of calcium ion-dependent cell surface glycoproteins that function in cell-cell adhesion. The cadherin family is divided into classical (Type I) and nonclassical (Type II) subtypes, as well as other categories which include protocadherins and cadherin-related molecules. The cadherin family is characterised by the presence of extracellular cadherin (EC) repeats within the ectodomain of the protein, which vary in number within the family. E-cadherin is a well-characterised single-pass transmembrane Type I cadherin that is primarily expressed on epithelial cells and contains a cytoplasmic domain of 150aa and an extracellular domain of 550aa containing five EC repeats, each of approximately 110aa [1, 2]. E-cadherin contributes to the generation and maintenance of adherens junctions (AJ) via homophilic (E-cadherin-E-cadherin interaction) and, most often, homotypic (epithelial-epithelial cell interaction) cell adhesion (Figure 1). This structure is likely to involve E-cadherin cis-homodimers binding similar cis-homodimers on adjacent cells to form transhomodimers, although the exact mechanism of this interaction is unclear [3]. Type I classical cadherins, which also include N-cadherin, P-cadherin, and VE-cadherin, possess a Histidine-Alanine-Valine (HAV) motif within the terminal EC repeat of the extracellular domain which is an essential cell adhesion recognition sequence [3]. Although there is some controversy surrounding the precise function of distinct regions of E-cadherin in cell-cell adhesion, many studies have shown the HAV domain, located on residues 79–81 of the EC1 domain, to play a key role in its adhesive function by forming a hydrophobic pocket into which a Tryptophan residue 2 (Trp2) from an adjacent E-cadherin molecule can dock. Mutations of Trp2 and the alanine residue of the HAV domain, W2A and A80I, respectively, have been shown to abolish trans- but not cis-homodimerisation of E-cadherin molecules, thus demonstrating the key roles of these amino acids in the formation of E-cadherin mediated cell-cell contact [2].

**Figure 2:**
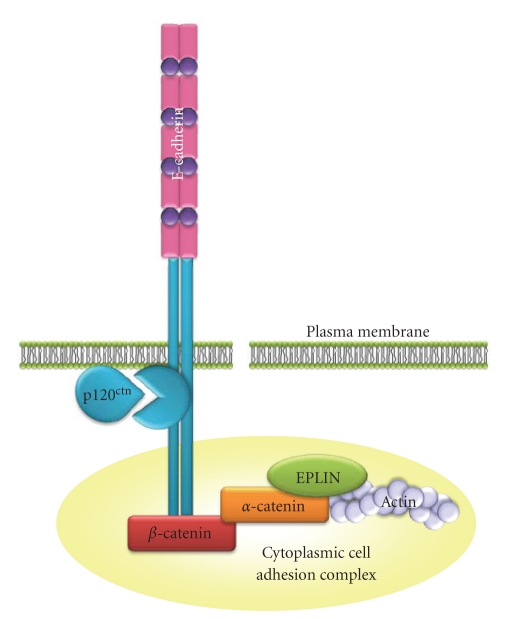
*E-cadherin and the Cytoplasmic Cell adhesion Complex.* E-cadherin is stabilised at the cell surface by its link to the actin cytoskeleton via *β*-catenin, *α*-catenin, and, possibly, Epithelial Protein Lost in Neoplasm (EPLIN). p120^ctn^ stabilises the CCC by preventing clathrin-mediated endocytosis.

**Figure 3:**
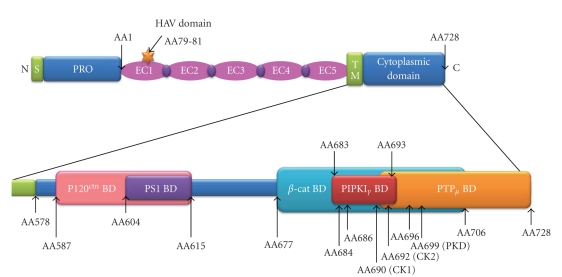
*Protein binding sites within the intracellular region of E-cadherin.* The cytoplasmic domain of E-cadherin contains binding sites for a variety of signalling molecules, thus facilitating its role in signal transduction. Abbreviations: S: signal peptide, PRO: propeptide, EC: extracellular domain, TM: transmembrane domain, N: N-terminus, C: C-terminus, *β*-cat: *β*-catenin, HAV: histidine-alanine-valine, BD: binding domain, PS1: presenilin 1, PIPKI*γ*: type I*γ* phosphatidylinositol phosphate kinase, PTP*μ*: protein tyrosine phosphate *μ*, AA: amino acid, CK: casein kinase, PKD: protein kinase D. Adapted from Van Roy and Berx [3].

The intracellular region of E-cadherin contains two conserved regions among the classical Type I and II cadherins, consisting of a juxtamembrane domain (JMD), also known as the membrane proximal cytoplasmic/conserved domain (MPCD), and a *β*-catenin binding domain. The *β*-catenin binding domain facilitates interaction of E-cadherin with the actin cytoskeleton via the Cytoplasmic Cell adhesion Complex (CCC), which consists of *β*-catenin, *α*-catenin, and, possibly, Epithelial Protein Lost In Neoplasm (EPLIN) [4] (Figure 2). The JMD facilitates binding of p120^ctn^ which stabilises the CCC by preventing clathrin-mediated endocytosis [5]. However, this convenient subdivision of the E-cadherin cytoplasmic domain (JMD and *β*-catenin domain) does not reflect the complexity of interactions within these two regions (Figure 3). For example, the JMD also binds Presenilin 1 which can inhibit p120^ctn^ binding and facilitate cleavage of the E-cadherin cytoplasmic domain (via *γ*-secretase) leading to disassembly of AJs. The *β*-catenin interacting region of E-cadherin also binds several other proteins. For example, the type I*γ* phosphatidylinositol phosphate kinase (PIPKI*γ*) binding domain lies within the *β*-catenin binding site [6]. PIPKI*γ* binds preferentially to dimerised E-cadherin and is responsible for the conversion of phosphatidylinositol phosphate (PIP) to phosphatidylinositol-4,5-bisphosphate (PIP_2_) [6]. Protein Tyrosine Phosphatase-*μ* interacts with the C-terminus of E-cadherin, partly overlapping the *β*-catenin binding domain, and is believed to protect E-cadherin from tyrosine phosphorylation [3].

## 2. Loss of E-Cadherin during Tumour Progression

Metastatic spread of tumour cells is the primary cause of death in cancer patients, with epithelial tumours representing at least 80% of all cancers. Loss of cell surface E-cadherin protein correlates with increased tumour cell invasion in the majority of epithelial tumours and is believed to impart epithelial-mesenchymal transition (EMT) properties to the cells, allowing increased motility and invasion [1, 7]. The role of E-cadherin as a metastasis repressor is well established [1, 8]. For example, loss of E-cadherin expression in epithelial cells leads to abrogation of cell-cell contact and increased motility [8, 9], whilst forced expression of E-cadherin protein in metastatic tumour cell lines is sufficient for reversal of this phenotype [1, 10]. E-cadherin is known to be regulated via several unrelated mechanisms. Repression of E-cadherin transcripts via E-box binding proteins (e.g., Snail and Slug) has been described in detail and is also associated with tumour cell metastasis [8, 11, 12]. MMP-7 and -13 can cleave cell surface E-cadherin protein resulting in a soluble ectodomain portion of E-cadherin protein that can act in a paracrine effect to inhibit E-cadherin function on neighbouring cells [13]. In addition, soluble E-cadherin fragments have been shown to induce MMP-2, MMP-9, and MMP-14 expression in lung tumour cells [14]. E-cadherin can also be internalised via the c-met receptor pathway following activation by HGF [15–17].

As well as loss of E-cadherin correlating with increased metastatic potential of epithelial-derived tumours, both *β*-catenin and *α*-catenin function as transactivating factors, the former by inhibiting TCF/LEF- and the latter by inhibiting Kaiso-induced repression of target genes [4, 5, 18]. Loss or aberrant expression of *α*-catenin is also associated with a malignant phenotype in many cancers [10, 11]. Initial studies by Watabe and colleagues [19] suggested that cadherin-catenin-mediated adhesion altered growth kinetics in a lung carcinoma cell line (PC9). Although these cells express E-cadherin and *β*-catenin, they do not express *α*-catenin and are unable to form cell aggregates when grown in suspension culture. However, upon transfection of *α*-catenin, E-cadherin-mediated cell-cell contact was restored and resulted in altered growth of these cells, indicating that E-cadherin adhesion may participate either indirectly or directly in cellular proliferation. Therefore, aberrant E-cadherin expression can also be induced by loss of function of cytoplasmic binding partners of the protein.

In addition to their structural role in cell-cell adhesion, many cadherins also participate in the transduction of signals from the cell membrane to the nucleus [1]. For example, N-cadherin has been shown to stimulate FGF signalling [20] whereas VE-cadherin acts as a coreceptor with VEGFR to facilitate TGF*β* signalling [21]. The dual involvement of *β*-catenin in formation of the CCC and Wnt signalling has led to the proposal of a mechanism implicating E-cadherin in Wnt signal transduction. In this model, E-cadherin sequesters *β*-catenin at the cell membrane to prevent Wnt-induced *β*-catenin/TCF transactivation [22, 23]. However, recent studies suggest that *β*-catenin exists in two separate functional compartments within the cell which function independently to maintain CCC integrity or facilitate Wnt-dependent transactivation [24]. The homophilic binding of E-cadherin that functions to maintain cell-cell adhesion can also regulate the action of the Rho family of GTPases via p120^ctn^ [25, 26]. For example, cadherin engagement has been shown to inhibit RhoA activity and activate Rac1 [27]. Rho-GTPases are small G-proteins that mediate cell motility and proliferation [1]; the dysregulation of which has also been implicated in tumorigenesis [28].

## 3. Embryonic Stem Cells

When cultured under appropriate conditions, embryonic stem (ES) cells possess the ability to self-renew indefinitely whilst retaining the pluripotent capacity to differentiate into any cell of the adult organism [29]. The pluripotency of human ES (hES) cells, shared with induced pluripotent stem (iPS) cells, provides enormous potential for their use in cell replacement strategies to target disorders that currently lack a long-term control strategy, such as type 1 diabetes [30]. In addition, the proliferative properties of these cells provide a useful model system to study self-renewal mechanisms that may be applicable to tumorigenesis.

### 3.1. An Epithelial-Mesenchymal Transition Event Occurs during ES Cell Differentiation

Throughout embryogenesis, cadherins play a key role in the sorting of heterogeneous cell populations to allow tissue segregation. The observation that E-cadherin null embryos are unable to form a trophectoderm epithelium or blastocoel is demonstrative of the crucial function of E-cadherin in embryo development [31]. The E-cadherin null mutation is embryonic lethal; however, derivation of E-cadherin^−/−^ mouse (m)ES cells from E-cadherin null embryos has allowed the critical role of E-cadherin in development to be dissected in more detail. EMT in epiblast cells allows their ingression within the primitive streak [1], and the morphological changes to these cells occur concomitantly with a shift from E-cadherin to N-cadherin expression at the cell surface [32].

We and others have shown that an EMT-like event occurs during ES cell differentiation [33–36]. Our data described an E- to N-cadherin switch during ES cell differentiation in monolayer culture which was associated with upregulation of the E-cadherin repressor proteins, Snail, Slug, and SIP1 [33, 34]. In addition, expression of MMP-2 and -9 transcripts was induced during this period which correlated with increased gelatinase activity and cellular motility [33, 34]. Therefore, differentiation of ES cells is associated with an EMT event that is similar to that observed during early embryogenesis. However, it should be noted that the process of EMT in ES cells is a predetermined event similar to that which occurs during early embryogenesis. In contrast, oncogenic EMT is likely to be a more complex and variable phenomenon. Indeed, the concept of oncogenic EMT remains a contentious issue since the study of this process during tumorigenesis in vivo is difficult, relying instead upon indirect observations or in vitro analysis of tumour cell lines which may not reflect the underlying physiology of the disease. Furthermore, there is recent evidence that tumour cells can spread in the absence of EMT [37, 38]. Thus, oncogenic EMT is unlikely to reflect a predetermined event and may well be influenced by the underlying genetics and age of the host, genetic instability of individual tumour cells, the organ in which the tumour originates, and the microenvironment. However, our studies in ES cells have allowed the function of loss of E-cadherin to be examined in detail. Below, we discuss our findings which demonstrate that loss of E-cadherin alone does not induce an EMT event in ES cells and relate this to observations in tumour cell lines in vitro. 

We investigated the function of E-cadherin and N-cadherin in mES cells by utilising knockout ES cell lines [34] or abrogation of E-cadherin function in hES cells using a neutralizing antibody (nAb). In both mES and hES cells, we observed that absence of E-cadherin activity resulted in loss of cell-cell contact and increased motility; however, the cells remained pluripotent and subsequent removal of the E-cadherin nAb led to reversion of the cells to a characteristic ES cell phenotype. Therefore, abrogation of E-cadherin-mediated cell-cell contact in ES cells can be a reversible event, as also observed in epithelial cell lines [39], which does not affect pluripotency of the cells. More importantly, abrogation of E-cadherin mediated cell-cell contact in both mES and hES cells did not induce a characteristic EMT-event, suggesting that loss of cell-cell contact alone is insufficient to promote EMT in these cells [33, 34]. We also demonstrated in mES cells that E- and N-cadherin are independently regulated during ES cell differentiation and the latter does not induce expression of EMT-associated transcripts and proteins, although absence of N-cadherin did significantly reduce cellular motility. Therefore, whilst cadherins are critical components of ES cell EMT, they do not directly regulate this process and loss of E-cadheirn alone is insufficient to induce such an event. Interestingly, this may also be the case in tumours of epithelial origin. For example, Andersen and colleagues [40] found that short-term inhibition of E-cadherin expression in A431 cells did not induce an EMT event. They suggested that the onset of EMT in tumour cells via functional inhibition of E-cadherin is a slow and gradual process which is associated with protracted genetic reprogramming of tumour cells. Therefore, studies in both ES and tumour cell lines suggest that loss of E-cadherin alone is insufficient to induce an EMT event. 

Loss of E-cadherin in ES cells, and other epithelial cells [39], can induce major changes in cellular architecture and localisation of plasma membrane-associated proteins. For example, abrogation of E-cadherin function in ES cells resulted in loss of cortical actin cytoskeleton arrangement and induction of cell polarization [33, 34]. Furthermore, we observed that in both mES and hES cells the trophoblast glycoprotein (5T4 antigen), which is a promigratory factor, was translocated from the cytoplasm to the plasma membrane in an energy dependent manner within 15 minutes of exposure of the cells to an E-cadherin nAb. Removal of the E-cadherin nAb from mES and hES cells resulted in restoration of cell-cell contact and absence of 5T4 antigen from the cell surface within 24 h. Interestingly, whilst forced expression of E-cadherin protein in E-cadherin^−/−^ ES cells restored cell-cell contact and reduced motility, the 5T4 antigen remained at the cell surface. 5T4 is a transmembrane glycoprotein that is upregulated on many carcinomas, and its expression correlates with poorer clinical outcome in ovarian, gastric, and colorectal cancers [41–45]. Forced expression of 5T4 in epithelial cells resulted in increased motility and loss of E-cadherin-mediated cell-cell contacts [46]. Therefore, our observations of 5T4 antigen and E-cadherin expression in ES cells is also reflected in epithelial cell lines.

We have also observed that loss of E-cadherin function in ES cells results in altered cell surface localisation of proteoglycans, which are important in basement membrane formation (Soncin et al., unpublished data). In addition, microarray analysis of E-cadherin^−/−^ ES cells revealed 2265 transcript alterations compared to wild-type (wt)ES cells, with effects confined not only to cell adhesion and motility but also affecting genes associated with primary metabolic processes, catabolism, apoptosis, and differentiation (Soncin et al., unpublished data). Therefore, our data suggests that the function of E-cadherin in ES cells is not merely to maintain cell-cell adhesion but also to regulate transcription associated with a diverse range of cell functions, maintain appropriate growth factor responsiveness of the cells, and retain plasma membrane localisation of a range of molecules. There are limited studies on the implication of loss of E-cadherin alone in normal epithelial cells in vivo or in vitro, and current evidence is predominantly histopathological analysis of tumour biopsies and in vitro analysis of tumour cell lines. Histopathological evidence for loss of E-cadherin in metastatic progression is well established; however, such analysis does not inform us of the molecular mechanisms underlying this process nor whether a true EMT event has occurred. In addition, most studies on loss of E-cadherin in tumour cell lines involve stimulation of EMT via exogenous compounds, such as Transforming Growth Factor-*β* [47], Interleukin-6 [48], Hepatocyte Growth Factor [49], and Tumour Necrosis Factor [50]. As such, there is limited evidence for the function of E-cadherin alone in normal epithelium. Furthermore, there is scant data assessing the expression of E-cadherin in early neoplasms, mainly due to difficulties of analysis in vivo. Therefore, the role of loss of E-cadherin in the formation and establishment of neoplasms is unclear. In addition, there is some debate as to whether neoplasms occur as a result of genetic/epigenetic alterations or whether these changes derive from selection of proliferating cells (see Somatic Mutation Theory and Tissue Organisation and Field Theory below). In our opinion, current theories of tumorigenesis do not provide sufficient explanation for the events leading to the establishment of a neoplasm nor the function of E-cadherin expression during this process. Since ES cells are karyotypically normal, they may afford a more appropriate model for studying the early stages of neoplasm formation within epithelium, and this is discussed later in this review.

### 3.2. E-Cadherin Regulates Growth Factor Signalling in ES Cells

**Figure 4:**
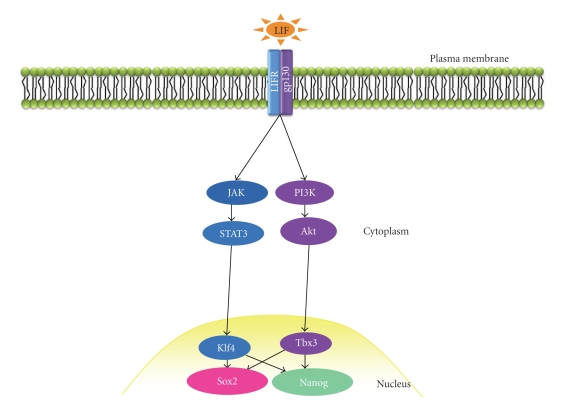
*LIF signal transduction in mouse ES cells.* Following the binding of LIF to the heterodimeric LIFR/gp130 complex, the Jak/STAT3 and PI3K/Akt signalling pathways are activated which regulate expression of Sox2 and Nanog via Klf4 and Tbx3, respectively.

In order to maintain pluripotency, mES cells require signals to inhibit differentiation (Figure 4). The first of these signals to be identified was leukaemia inhibitory factor (LIF [51]), an interleukin-6 family cytokine that binds a heterodimeric complex of gp130 and the LIF receptor *β* subunit (LIFR). Gp130 is activated upon LIF engagement, triggering a number of signal transduction networks including the Janus kinase (Jak)/Signal Transducer and Activator of Transcription 3 (STAT3) pathway and the PI3K/Akt cascade [52, 53]. The Jak/STAT3 and PI3K/Akt pathways have recently been linked to components of the “core circuitry” of pluripotency, sex determining region y-box 2 (Sox2), and Nanog proteins, via Krüppel-like factor-4 (Klf4) and the T-box transcription factor Tbx3 [52] (Figure 4). Bone morphogenetic proteins (BMPs) present within serum in the culture medium were later shown to inhibit neuroectoderm lineage specification, with mES cells shown to self-renew in serum-free medium containing LIF, Bmp4, and N2/B27 supplements [54]. It has subsequently been demonstrated that mES cells can be cultured in the absence of LIF and Bmp4 in medium supplemented with antagonists/agonists of the FGF, ERK, and Wnt pathways [55]. 

**Figure 5:**
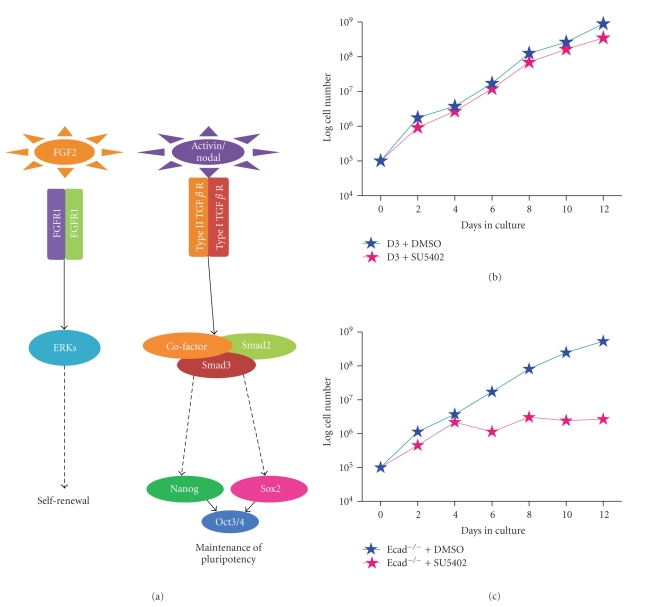
*Fgf2 and Activin/Nodal signal transduction in mouse E-cadherin^−/−^ ES cells.* (a) E-cadherin^−/−^ ES cells maintain pluripotency via Activin/Nodal signalling and self-renewal through Fgf2 signalling. (b) Wild-type ES cells treated with the FGFR1 small molecule inhibitor SU5402 exhibit similar proliferation rates compared to control-treated (DMSO) cells. (c) E-cadherin^−/−^ ES cells treated with the FGFR1 small molecule inhibitor SU5402 exhibit significantly reduced proliferation rates compared to control-treated (DMSO) cells.

Whilst E-cadherin^−/−^ ES cells can be cultured in vitro as pluripotent cells [34] in media supplemented with foetal bovine serum (FBS) and LIF, we later discovered that the cells do not utilise LIF under these conditions [56]. Instead, E-cadherin^−/−^ ES cells maintain pluripotency via the Activin/Nodal pathways whilst optimal proliferation (self-renewal) is achieved via Fibroblast growth factor-2 (Fgf-2) (Figure 5(a)). Addition of an FGFR1 inhibitor (SU5402) to wild-type (Figure 5(b)) or Ecad^−/−^ ES cells (Figure 5(c)) demonstrated the reliance of the latter cells for self-renewal via this pathway. The presence of Activin, Nodal, and Fgf2 in the FBS used for ES cell culture is likely to reflect the ability of E-cadherin^−/−^ ES cells to self-renew in the absence of LIF. Further analysis of E-cadherin^−/−^ ES cells demonstrated that these cells could proliferate in serum-free medium supplemented with Activin/Nodal and Fgf-2, with exposure to SB431542, an inhibitor of Activin-like kinase receptors (Alks)-4, -5, and -7, inducing loss of the pluripotency markers Oct4 and Nanog [56]. Forced expression of full-length E-cadherin in E-cadherin^−/−^ ES cells restored cell-cell contact and LIF-dependent self-renewal via Stat3 signalling [56]. Reversible Activin/Nodal-mediated pluripotency was also observed in wtES cells treated with an E-cadherin homodimerisation-inhibiting peptide, CHAVC, which is likely to target the HAV domain or Trp2. Interestingly, cells treated with the E-cadherin nAb DECMA-1 did not exhibit LIF-independent pluripotency, suggesting that specific regions of E-cadherin protein regulate this effect and it is not simply due to loss of cell-cell contact. Furthermore, Ecad^−/−^ ES cells can also maintain pluripotency in serum free medium supplemented with LIF, Bmp4, and N2/B27 demonstrating that these cells possess a functional “ground state” pluripotent signalling pathway (as described by Ying et al. [57]), as well as the ability to circumvent this pathway by utilising Activin and Nodal. Further evidence for the role of E-cadherin in mES cell self-renewal has been demonstrated in FAB-SCs, mouse stem cells derived using Fgf2, Activin, and BIO [58]. In this study, FAB-SCs exhibited limited chimaerism, but when cultured in LIF-containing medium, this was restored and subsequent repression of E-cadherin in these cells induced differentiation. Therefore, E-cadherin functions in ES cells to regulate pluripotency via Jak/Stat3 signalling. It has been reported that Stat3 can be activated through homophilic interactions of E-cadherin in mouse mammary epithelial cell lines [59]. In this study, the authors plated cells onto surfaces coated with fragments encompassing the two outermost domains of E-cadherin and demonstrated activation of Stat3, even in the absence of direct cell-to-cell contact. Therefore, regulation of Stat3 signalling pathways by E-cadherin has been demonstrated in both ES and epithelial cells.

To investigate the region of E-cadherin responsible for LIF-dependent pluripotency in mES cells, we utilised cDNA exhibiting truncated regions of the E-cadherin cytoplasmic domain and expressed the protein in E-cadherin^−/−^ ES cells. E-cadherin^−/−^ mES cells expressing E-cadherin lacking the terminal 71 amino acids of the cytoplasmic region, which includes the *β*-catenin binding domain, maintained pluripotency via the Activin/Nodal pathway whereas wild-type E-cadherin protein restored LIF-dependent pluripotency [56]. This data suggests that the E-cadherin/*β*-catenin complex is responsible for LIF-dependent pluripotency of mES cells. This conclusion is corroborated by the observation that *β*-catenin null mES cells also exhibit Activin/Nodal-mediated self-renewal, irrespective of E-cadherin protein expression [56]. 

In human ES cells, TGF*β* family signalling has been shown to be critical for maintenance of pluripotency and self-renewal (Figure 5(a)). When bound to their dimeric Activin-like kinase (Alk) receptors, Activin and Nodal initiate a signalling cascade involving the phosphorylation of Smads 2 and 3 which subsequently form a complex with Smad4 allowing translocation to the nucleus, cofactor binding, and activation of target genes [60], including Nanog [61]. Specifically, Activin/Nodal signalling via Smad2/3 is required to maintain hES cell pluripotency, with FGF2 acting as a competence factor for Activin/Nodal signal transduction [62]. This is similar to our observations in E-cadherin^−/−^ ES cells, suggesting that E-cadherin protein expression levels function to determine pluripotent signalling pathways in ES cells.

Mouse ES cells lacking a functional E-cadherin/*β*-catenin complex, therefore, resemble the self-renewal properties of hES cells, FAB-SCs, and mouse epiblast-derived stem cells (EpiSCs). Interestingly, E-cadherin has been shown to be downregulated in EpiSCs in comparison to wtES cells [63]. It therefore appears that low levels of E-cadherin in mouse-derived pluripotent cells correlate with Activin/Nodal-mediated self-renewal whereas higher levels of expression are associated with LIF-dependent maintenance of pluripotency (Jak/Stat3). We have observed that partial RNA interference (RNAi) of E-cadherin in mouse ES cells also results in LIF-independent pluripotency [56]. Nagaoka and colleagues [64] have demonstrated that E-cadherin-coated tissue culture plates can decrease the dependence of mES cells to LIF-dependent self-renewal, although the cells could not be grown in the absence of this cytokine. We have also observed that culture of hES cells in the presence of the E-cadherin nAb SHE78.7 allows culture of the cells in the absence of FGF-2, even where cell-cell contact is not completely inhibited (Patent WO2007088372). Therefore, total abrogation of E-cadherin-mediated cell-cell contact in ES cells may not be necessary for altered growth factor response in these cells, although the underlying mechanisms remain unknown.

## 4. The Role of E-Cadherin in Regulating Growth Factor Response during Tumorigenesis and the Relationship to Observations in ES Cells

In order for a cell to become cancerous, it must undergo a series of cellular alterations resulting in increased replicative potential. Such growth independence may be attributed to mechanisms in which regulatory pathways are perturbed and can occur at differing levels of signal transduction. Alterations in growth factor signalling are a predominant feature of tumour progression; tumour cells secrete elevated levels of growth factors, which substitute for exogenous growth factor requirements, or become resistant to physiologically inhibitory exogenous growth factors. Furthermore, altered expression at the receptor level or deregulation at the level of secondary messengers may also contribute to tumorigenesis [65, 66]. Whilst there are a plethora of growth factors associated with tumorigenesis, for the purposes of this review we will focus on growth factors that have been associated with E-cadherin expression.

### 4.1. Receptor Tyrosine Kinase Family

The ErbB family of receptor tyrosine kinases (RTKs) are important in maintaining normal epithelial cell function. RTKs are a diverse family of receptors that include, amongst others, Epidermal Growth Factor Receptor (EGFR), Fibroblast Growth Factor Receptors (FGFR), Vascular Endothelial Growth Factor Receptor (VEGFR), and Ephrin (Eph) receptors and are critical signalling components of embryonic development and adult homeostatic functions [67, 68]. Consequently, their role in growth factor receptor signalling has resulted in numerous RTKs being implicated in multiple malignancies by overexpression of ligand receptors [69]. In particular, the expression or activation of EGFR is altered in many epithelial tumours [70], and both EGFR and ErbB2 are validated targets for cancer chemotherapeutics that are in current use for treatment of breast, lung, colorectal, and head and neck cancers [71, 72]. Qian et al. [73] first demonstrated that E-cadherin was able to inhibit activation of EGFR in epithelial cells, demonstrating a bidirectional relationship between E-cadherin and EGFR. A recent study using recombinant cadherin ligand assays showed that E-cadherin homophilic interactions specifically inhibited EGFR signalling by disrupting the STAT5b signalling pathway [74]. These data suggest that E-cadherin is able to negatively regulate mitogenic signalling in tumours mediated by EGFR and that E-cadherin may have an inhibitory effect on numerous RTKs, a phenotype observed in many tumours [73, 75, 76]. The dynamic relationship between E-cadherin and EGFR is interesting since EGFR expression is believed to be an early event during tumourgenesis [75], whereas E-cadherin downregulation has been previously associated with later stages (e.g., EMT). 

Utilising E-cadherin^−/−^ ES cells, we have shown that abrogation of E-cadherin expression alters the cellular response to the microenvironment and increases proliferation [56]. Unpublished global gene array analysis of E-cadherin^−/−^ ES cells in our lab has revealed that a significant proportion of the “top 20” upregulated genes in these cells are RTKs (Soncin et al., manuscript submitted). For example, both EphA1 and EGFR transcripts are amongst the “top ten” upregulated genes in E-cadherin^−/−^ ES cells compared to the parental cell line. The temporal regulation of EGFR expression during early stages of tumorigenesis and its expression following loss of E-cadherin in ES cells supports our hypothesis (described below) that aberrant regulation of E-cadherin in epithelial cells alters their response to exogenous growth factors, resulting in autonomous cell growth and neoplasm formation in the absence of EMT.

### 4.2. Transforming Growth Factor *β* Family

Transforming growth factor *β* (TGF*β*) signalling is central to many cellular processes such as cell cycle arrest, angiogenesis, and homeostasis, and, as such, its subsequent role in tumorigenesis and invasion is complex. TGF*β* signal transduction is mediated via TGF*β*1, -*β*2, -*β*3, Activin, and Nodal. These ligands bind to a cell surface receptor complex consisting of a pair of serine/threonine kinases, TGF*β* receptor type I (TGF*β*R1), and type II (TGF*β*R2) [77]. The signal is further propagated through phosphorylation of Smad proteins. There is significant evidence demonstrating a dual role for TGF*β* signalling in both promotion and suppression of tumorigenesis in a variety of malignancies [78–82]. Of interest is the role of TGF*β* signalling in a subset of cells that possesses increased tumourigenic capacity. Recent evidence suggests that this specific cell population exhibit many features typical of stem cells, such as self renewal and multipotency, and have been termed cancer stem cells (CSCs). Activin receptors exhibit altered expression in cancers [83], and mice deficient in Inhibin-*α* (an activin antagonist) develop tumours within four weeks of birth [84]. Microarray analysis of E-cadherin^−/−^ ES cells has revealed a number of growth factors and their receptors that are altered as a consequence of loss of E-cadherin (Soncin et al., unpublished data) and that these growth factors and their receptors are similarly altered in a significant number of tumour types. For example, BMP4, TGF*β*1, and Inhibin-*β* B are found in the “top 10” genes downregulated in response to abrogation of E-cadherin compared to wtES cells.

### 4.3. Fibroblast Growth Factor Family

Studies by Halaban and colleagues [85] first demonstrated a role for autocrine FGF signalling in tumorigenesis. Melanomas were found to express high levels of FGF2 and FGFR1, and inhibition of expression of either of these molecules resulted in inhibition of tumour cell growth and progression [86], similar to that observed in mouse E-cadherin^−/−^ ES cells (Figure 5(b)) and hES cells. Moreover, extracellular FGF2 expression contributes to radio- and chemotherapy resistance in multiple tumour types, further validating the importance of the tumour cell microenvironment in tumorigenesis [87–89]. Ruotsalainen et al. [90] reported elevated levels of FGF2 in serum of small cell lung cancer patients, which correlated with poor prognosis and active angiogenesis, and elevated expression of FGF2 (amongst others) has been detected in breast and prostate malignancies [91, 92]. Human ES cells are dependent upon exogenous FGF2 to maintain pluripotency *in vitro *[93], and, in mES cells lacking E-cadherin, Fgf2 is necessary for self-renewal [56]. FGF5 is expressed in embryonic tissues but scarcely detected in adult tissue; however, expression of FGF5 and its receptor are associated with malignancy in astrocytic brain tumours [94]. Although to date there is no evidence to suggest that E-cadherin affects FGF5 expression in cancer cells, we have shown that transcripts for this protein are significantly upregulated in mouse E-cadherin^−/−^ ES cells compared to wtES cells [56]. This may indicate that the abnormal expression of FGF5 in cancer cells may be due to alterations in E-cadherin expression in these cells. 

In summary, we have shown that growth factors and their receptors associated with tumorigenesis appear to be regulated by E-cadherin expression in a similar manner in epithelial, tumour-derived, and ES cells. In the following section, we present a hypothesis that dysregulation of E-cadherin in epithelial tissues is a determining event in altering growth factor response of the cells leading to neoplasm formation and subsequent tumorigenic phenotype in the absence of EMT.

## 5. Models of Tumorigenesis

Three hypotheses have gained significant interest in attempting to explain events leading to tumorigenesis. The Somatic Mutation Theory (SMT) considers tumorigenesis to be a multistep evolutionary process where specific mutations confer a selective proliferative advantage to a normally quiescent cell. By contrast, the Tissue Organisation Field Theory (TOFT) suggests that tumorigenesis reflects organogenesis “gone awry”, due to tissue disorganisation. The Cancer Stem Cell Hypothesis (CSCH) suggests that tumorigenesis results from abnormal proliferation of stem cells leading to differentiated transit amplifying cells (TACs) making up the bulk of the tumour cell mass. SMT relies upon individual cells exhibiting a default state of quiescence with mutations in regulatory genes inducing cell proliferation. TOFT is the antithesis, where cells possess a default state of proliferation which is controlled by the microenvironment, and, even where mutations are present, cells will remain established within a normal tissue until abnormal tissue organisation occurs.

### 5.1. Somatic Mutation Theory

SMT remains the prevailing model for the occurrence of sporadic tumours, which account for around 95% of all cancers [95]. The theory suggests that sporadic tumour formation derives from multiple DNA mutations within a single somatic cell and the subsequent progeny proliferate to form the tumour mass. As such, this model dictates that tumorigenesis is the result of abnormal somatic cell proliferation achieved by mutations of genes governing cell cycle and proliferation. Whilst this simple model has many advocates, subsequent research has gradually undermined some of the core principles of this theory. For example, the low occurrence of genetic mutations observed in somatic cells has questioned the relevance of the SMT model to tumorigenesis since these cannot explain the high numbers of mutations found in neoplasms [96]. In addition, the isolation of embryonal carcinoma (EC) cells, derived from teratocarcinomas, has further questioned the prerequisite of genetic mutations for tumorigenesis. For example, some EC cell lines, which are the stem cells of teratocarcinomas, can incorporate normally within the tissues of mice [97, 98]. Normal function of these chimaeric mice is dependent upon a low level of EC chimerism, demonstrating that embryo-derived, karyotypically normal cells can negatively regulate the proliferative and malignant phenotype of EC-derived somatic cells. Whilst these observations do not disprove SMT, they do illustrate that genetic mutations may not be the primary reason for tumorigenesis in teratocarcinomas. Thus, the tissue microenvironment is likely to play a major role in regulating mutated cells to maintain normal tissue homeostasis.

### 5.2. Tissue Organisation and Field Theory

TOFT has been developed by Sonnenschien and Soto [99] and consists of two default premises: (1) tumorigenesis is a problem of tissue organisation, comparable to organogenesis during early development and (2) proliferation is the default state of all cells. TOFT suggests that carcinogens affect stromal cells which subsequently results in changes in the microenvironment and abnormal organisation of the epithelium, leading to default proliferation of the cells. In this respect, the presence of mutations within an epithelial cell will not result in formation of a neoplasm until disorganisation of the epithelium has occurred. Indeed, the thesis behind TOFT is that carcinogenesis is a “community effect” rather than a single “cell effect.”

### 5.3. Cancer Stem Cell Hypothesis

Conventionally, tumours were viewed according to the principles of the stochastic model; in that all cells of the tumour were equal in their proliferative ability and contribution to tumour spread. Moreover, the clinical implication of this model is that to successfully treat a tumour all of the cells need to be removed. The embryonal rest theory of cancer was first proposed by Virchow in 1855 [100–103], suggesting that tumours arise from dormant embryonic-like cells that maintain their tumorigenic capacity. This theory is similar to the current CSCH which, in the last decade, has revealed new insights in tumour biology by applying the principles of stem cell biology [80]. Original studies by Lapidot et al. [104] retrospectively identified the presence of a subpopulation of cells with a distinct phenotype and functionality in acute myeloid leukemia. These cells exhibited markers associated with normal hematopoietic stem cells and had clonogenic ability upon injection into athymic mice [105]. Subsequent publications have since shown that such cancer stem cells (CSCs), or side population cells (a semipurified group of cancer cells that contain a proportion, but not solely consisting of, CSCs), have been identified in many malignancies including breast, neck, blood, and colon [104, 106–108]. By consensus definition, a CSC is a cell within the tumour that possesses the capacity to self-renew and to produce the heterogeneous lineages of cells that comprise the tumour [109]. Further evidence for the CSCH can be observed from the heterogeneity within a tumour, which is retained by its metastases [105]. This indicates that the cell(s) responsible for secondary tumours possess a multi-differentiative capacity, a feature of stem cells. However, how does this minor population of cells (typically 0.1-0.2% of the tumour cell mass) support tumour growth without being “diluted out” by the tumour cells themselves? Kelly et al. [110] and Yoo and Hatfield [111] proposed that upon syngeneic transplantation of mouse leukemias a much larger proportion of the cells contributed to tumour propagation and that dominant clones, and not rare CSCs, may sustain many tumours.

### 5.4. Dysregulation of E-Cadherin and Formation of a Stem Cell-Like Phenotype

Independent experimental evidence has suggested that induction of an EMT-event in immortalised human mammary epithelial cells (HMECs) results in acquisition of a stem cell-like phenotype [112], with multipotency of the cells similar to that observed in mesenchymal stem cells. EMT was induced in HMECs by ectopic expression of Snail, Twist, or TGF*β* leading to increased invasion and migration of the cells. However, since induction of EMT in HMECs will result in altered E-cadherin expression, it is possible that loss of E-cadherin-mediated growth factor response of the cells may reflect these observations, rather than the EMT event itself.

## 6. Dysregulation of E-Cadherin in Neoplasia and Tumorigenesis (DENT) Hypothesis

Below, we discuss our observations of the function of E-cadherin in ES and somatic epithelial cells in the context of tumorigenesis to propose a hypothesis termed Dysregulation of E-cadherin in Neoplasia and Tumorigenesis (DENT). The DENT hypothesis should not be viewed as an alternative to current tumorigenesis hypotheses but more as an additional component of CSCH that attempts to explain events occurring during the early stages of neoplasia formation. Our aim is for the DENT hypothesis to stimulate debate regarding mechanisms associated with neoplasia formation and subsequent establishment of a tumour cell mass. We suggest that aberrant E-cadherin expression in epithelial cells is a decisive factor in the establishment of a neoplasm by altering growth factor response in the *absence* of EMT. We employ the term “aberrant E-cadherin expression” to include, amongst others, transcript repression and protein degradation as well as loss of structural integrity via loss of binding of E-cadherin to the actin cytoskeleton (i.e., altered *β*-catenin, *α*-catenin, p120^ctn^, or EPLIN expression). We propose that aberrant E-cadherin expression in an epithelial cell(s) results in altered growth factor response allowing the cells to circumvent existing microenvironment growth factor regulation and, instead, respond to exogenous or endogenous factors that stimulate proliferation and inhibit apoptosis. In addition, aberrant E-cadherin expression may result in transition of the cells into a stem cell-like phenotype. We suggest that the correlation between loss of E-cadherin and a more aggressive tumour phenotype *in vivo *reflects a requirement for the cells to escape growth factor responses that are inhibitory to cell growth and proliferation, rather than increased cellular motility *per se*. Therefore, we propose that aberrant regulation of E-cadherin in epithelial cells leads to long-term maintenance of a proliferative cancer stem cell-like phenotype and, as described by Andersen and colleagues [40], results in protracted genetic reprogramming of the cells subsequently leading to EMT and metastasis in later stages of the disease.

### 6.1. Evidence for Loss of E-Cadherin in Promoting Neoplasms

Forced expression of E-cadherin in the gut epithelium leads to decreased proliferation and increased apoptosis of epithelial cells [113], suggesting that E-cadherin functions to maintain epithelial integrity by negatively regulating abnormal cellular growth. In addition, expression of N-cadherin instead of E-cadherin in the intestinal epithelium of mice resulted in hyperproliferation of epithelial cells, decreased apoptosis, and neoplastic formations in the intestinal crypts [114]. This phenotype was associated with increased Wnt activity and loss of BMP signalling within the intestine; the latter of which is similar to that observed in E-cadherin^−/−^ ES cells. Whilst Libusova and colleagues [114] regarded this observation to be a specific result of N-cadherin expression, this effect may also reflect absence of E-cadherin in the intestinal epithelium. Therefore, these observations provide evidence for the role of loss of E-cadherin in neoplasm formation. We have also observed that inhibition of E-cadherin expression in ES cells results in increased proliferation of the cells [56] (Mohamet, unpublished data in hES cells). It is possible that increased proliferation of epithelial cells, following aberrant E-cadherin expression, leads to *de novo* mutation via selective adaptation. Therefore, it is feasible that some neoplasms can occur in the absence of inherent mutations, as observed by Libusova and colleagues [114]. However, for the purpose of this review, we will assume that epithelial cells already possess the prerequisite genetic mutations associated with tumorigenesis.

The DENT hypothesis will be discussed below in the following key stages of tumorigenesis:

1. neoplasm formation,
2. establishment of a tumour cell mass,
3. EMT and metastasis.

**(1) Neoplasm Formation**

**Figure 6:**
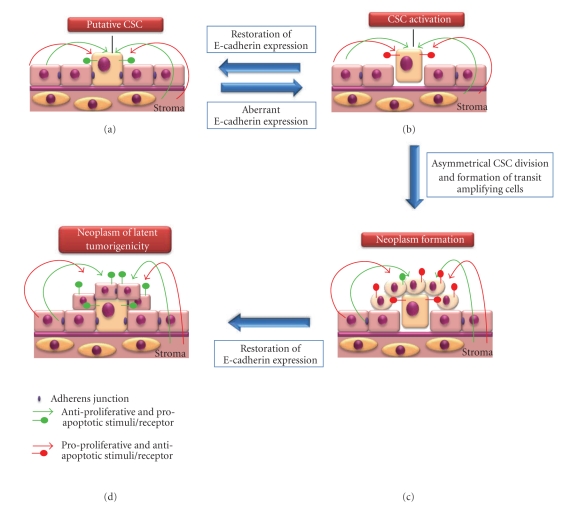
*Aberrant E-cadherin expression and neoplasm formation.* (a) E-cadherin functions in epithelial cells to enable recognition and responsiveness to antiproliferative and proapoptotic signals (shown by green arrows and receptors) and repression of recognition and responsiveness to proproliferative and antiapoptotic signals (shown by red arrows). (b) Upon dysregulation of E-cadherin expression the epithelial cell circumvents antiproliferative and proapoptotic signal regulation and, instead, responds to proproliferative and antiapoptotic stimuli, if present (shown by red receptors on the cell). For simplicity, such a cell is referred to as a cancer stem cell (CSC). At this point the CSC may exhibit restoration of E-cadherin expression, re-establish within the epithelium and lose its stem cell-like phenotype (i.e., return to the state shown in (a)). (c) The CSC exhibits asymmetric self-renewal leading to formation of TACs which, due to dysregulation of E-cadherin, fail to participate in normal tissue formation and, instead, form a neoplasm. (d) The CSC and TACs may exhibit restoration of E-cadherin expression allowing their reestablishment within the epithelium, resulting in a neoplasm of latent tumorigenicity (NLT).

The first stage of tumorigenesis is the formation of a neoplasm, the abnormal proliferation of cells. We propose that any epithelial cell has the potential to form a neoplasm; however, this process is inhibited within normal epithelium by the expression of E-cadherin.  Figure 6(a) shows that E-cadherin functions in epithelial cells to enable recognition and responsiveness to antiproliferative and proapoptotic signals (shown by green arrows and receptors) and repression of recognition and responsiveness to proproliferative and antiapoptotic signals (shown by red arrows). Thus, expression of E-cadherin in epithelial cells maintains epithelial integrity via appropriate growth factor recognition and responsiveness. Upon dysregulation of E-cadherin expression, perhaps via tissue damage, the epithelial cell circumvents antiproliferative and proapoptotic signal regulation and, instead, responds to proproliferative and antiapoptotic stimuli, if present (Figure 6(b), shown by red receptors on the cell). At this point, the cell may revert to normal E-cadherin expression and reestablish within the epithelium (Figure 6(a)). Alternatively, the cell may transform into a stem cell-like phenotype, leading to formation of TACs which, due to dysregulation of E-cadherin, fail to participate in normal tissue formation and, instead, form a neoplasm (Figure 6(c)). For clarity, we will term a cell exhibiting stem cell-like properties a “CSC”, although acquisition of this phenotype may be a protracted process. We further suggest that in early stages of neoplasia (Figure 6(c)), aberrant E-cadherin expression is reversible, and, where normal E-cadherin expression is restored to the CSC, it will reestablish within the epithelium, lose its stem cell-like phenotype, and form a neoplasm of latent tumorigenicity (NLT) (Figure 6(d)). In this scenario, a further event that induces aberrant E-cadherin expression would be required to resume further neoplastic tissue growth and, until this event occurs, the cells could persist within the epithelium without pathological consequence and maintain normal epithelial integrity. It is important to note that complete loss of E-cadherin expression in epithelial cells may not be necessary to elicit an altered growth factor response. For example, we have observed that partial knockdown of E-cadherin in ES cells is sufficient to induce altered growth factor response in these cells [56].

**Figure 7:**
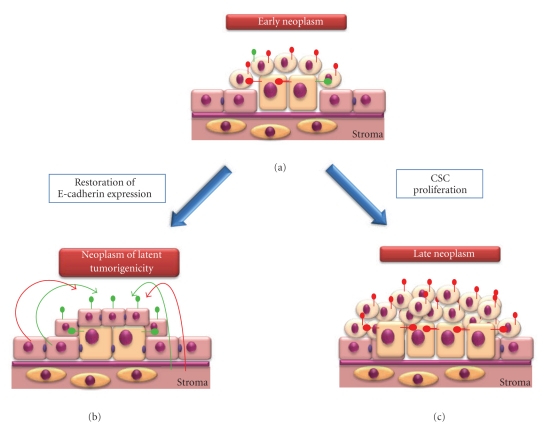
*Aberrant E-cadherin expression and formation of a late stage neoplasm.* (a) Symmetrical CSC division within the early stage neoplasm results in multiple CSCs within the neoplastic population. (b) Restoration of E-cadherin expression within the CSCs and TACs leads to their reestablishment within the epithelium, loss of stem cell-like properties and formation of a neoplasm of latent tumorigenicity. (c) Alternatively, cellular proliferation continues unabated resulting in a late stage neoplasm comprising CSCs and TACs.

Whilst differentiated TACs are believed to form the bulk of a tumour cell mass, there are many reports demonstrating the isolation of stem cell-like cells from solid tumours. Often, these stem cell-like cells, termed CSCs, are isolated as a side population (SP) from dissociated tumours [106–109], and rarely represent more than 1% of the total tumour cell population. The observation that CSCs can be isolated from many tumours suggests that these cells must exhibit proliferation to maintain their presence within the tumour cell mass. The occurrence of multiple CSCs within a tumour derived from a single CSC can be explained by (1) symmetrical self-renewal of the CSC or (2) dedifferentiation of TACs into a CSC-like phenotype. Symmetrical self-renewal of neural stem cells has been shown, where a combination of Fgf-2 and Egf induced niche-independent proliferation of the cells [115]. In addition, a capacity for limited symmetrical self-renewal of breast stem cells has also been described [116]. Irrespective of the mechanism responsible for formation of multiple CSCs within a population (Figure 7(a)), we suggest that these cells can also re-establish within the normal epithelium to form a NLT (Figure 7(b)). Where this does not occur, cellular proliferation continues unabated resulting in a late-stage neoplasm formed of the CSCs and TACs (Figure 7(c)). Therefore, in our model, neoplastic tissue formation is a reversible event and this may explain the occurrence of benign neoplasms (NLTs) within the epithelium.

**(2) Establishment of a Tumour Cell Mass**

**Figure 8:**
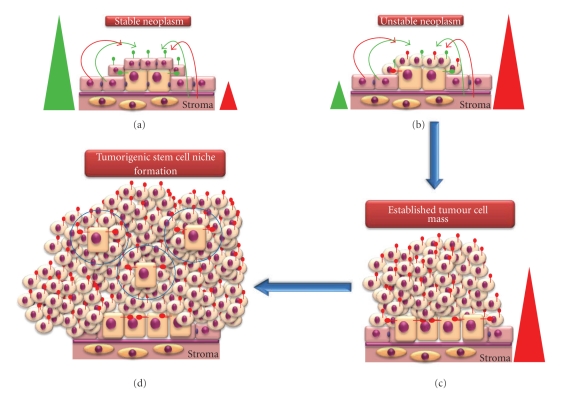
*Establishment of a tumour cell mass from an early stage neoplasm.* (a) Where the ratio of antiproliferative/proapoptotic (green triangle) to proproliferative/antiapoptotic (red triangle) signals/receptors is high, then a tumour will fail to establish and will remain as a stable neoplasm (e.g., NLT). (b) An unstable neoplasm forms where the ratio of antiproliferative/proapoptotic to proproliferative/antiapoptotic signals/receptors is low and the microenvironment is no longer capable of positively regulating tissue homeostasis. (c) When the microenvironmental signals are tipped in favour of aberrant E-cadherin expression (i.e., a proproliferative/antiapoptotic phenotype), the neoplasm becomes an established tumour cell mass due to proliferation of the CSCs and TACs. (d) Reorganisation of the tumour cell mass leads to the formation of new tumorigenic stem cell niches (shown in blue circles) which subsequently regulate CSC proliferation.

We have already discussed that some EC cell lines can incorporate and function normally within the tissues of chimaeric mice [97, 98], although this appears to be dictated by the ratio of normal to EC-derived cells within the animal. For example, where the ratio of normal- to EC-derived cells is high, then tissue homeostasis is maintained. However, where this ratio is low, the microenvironment appears unable to negatively regulate EC-derived cellular proliferation, resulting in tumorigenesis. This is likely to reflect the ratio of antiproliferative/proapoptotic to proproliferative/antiapoptotic signals within the microenvironment and the levels of appropriate receptors on the cells. We expand this observation to our hypothesis and suggest that where the ratio of antiproliferative/proapoptotic to proproliferative/antiapoptotic signals and receptors is high, then a tumour mass will fail to establish and will remain as a stable neoplasm (Figure 8(a)). However, where the ratio of antiproliferative/proapoptotic to proproliferative/antiapoptotic signals and receptors is low, the microenvironment is no longer capable of positively regulating tissue homeostasis and the neoplasm becomes unstable (Figure 8(b)), with the potential for establishment of a tumour cell mass (Figure 8(c)). It is likely that once this equilibrium is tipped in favour of aberrant E-cadherin expression (i.e., a proproliferative/antiapoptotic phenotype), then the neoplasm becomes an established tumour mass due to proliferation of the CSCs and TACs. Furthermore, increased proliferation of CSCs may subsequently lead to new tumorigenic stem cell niches (TSCN) being formed from TACs and their progeny which subsequently regulate CSC proliferation (Figure 8(d)). This scenario explains the isolation of CSCs from established high cellular mass tumours, where the expansion of the tumour cell mass implies the presence of proliferative CSCs. At this point (Figure 8(d)), dysregulation of E-cadherin expression is likely to be largely irrelevant to tumour cell growth since expression of this protein will be under sole control of the TSCN, and E-cadherin expression may well be required for establishment and maintenance of the TSCN. Indeed, the established tumour cell mass is likely to contain both E-cadherin-positive and -negative cells, with its regulation and expression under control of the TSCN. Thus, progressive loss of E-cadherin within a tumour should not be viewed solely as a consequence of metastatic potential but also in the formation of a neoplasm and the early events leading to the establishment of a tumour cell mass.

**(3) EMT and Metastasis**

**Figure 9:**
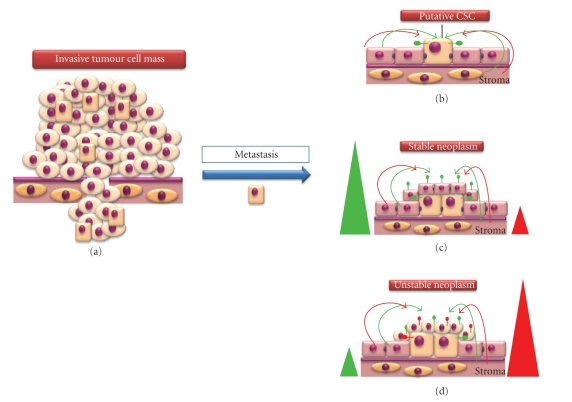
*Metastasis of CSCs from an invasive tumour.* (a) An invasive tumour cell mass results in metastasis of CSCs. (b) A CSC at a secondary site may exhibit restoration of E-cadherin expression leading to establishment within the epithelium. (c) The CSC may divide asymmetrically and symmetrically resulting in TACs and CSCs, however, where the ratio of antiproliferative/proapoptotic (green triangle) to proproliferative/antiapoptotic (red triangle) signals/receptors is high, then a stable neoplasm will form. (d) Alternatively, the pioneer CSC may maintain aberrant E-cadherin expression resulting in a low ratio of antiproliferative/proapoptotic to proproliferative/antiapoptotic signals/receptors and formation of an unstable neoplasm with potential for establishment of a tumour cell mass.

As we have demonstrated in ES cells, and by Andersen and colleagues [40] in A431 cells, loss of E-cadherin alone is insufficient to induce an immediate EMT event; therefore, aberrant E-cadherin expression in a tumour cell will not necessarily induce invasion and metastasis. However, absence of E-cadherin will result in altered growth factor response, and this may increase the likelihood of cells responding to exogenous or endogenous factors that can stimulate expression of EMT-associated molecules, such as MMPs, as well as gradual genetic reprogramming of the cells [40]. Thus, aberrant E-cadherin expression within a tumour cell mass is likely to lead to intensification of the metastatic phenotype. For example, it has been shown that soluble extracellular E-cadherin fragments can induce a positive feedback loop of gelatinase expression in lung tumour cells [14]. We have already discussed the importance of E-cadherin in regulating epithelial integrity, and it is likely that a metastatic cell will be dependent on E-cadherin expression for establishment at a secondary site. This is corroborated by experimental data showing that secondary tumours derived from carcinomas often contain cells within the population expressing E-cadherin [117–119]. Therefore, it is possible that “successful” metastatic cells will retain control of E-cadherin regulation rather than exhibiting irreversible epigenetic silencing or mutation of this gene. This suggests that “successful” metastatic cells are likely to be CSCs in which E-cadherin regulation is maintained (Figure 9). Indeed, it is possible that E-cadherin expression within a metastatic CSC allows its establishment within the secondary site and that the process of dysregulation of E-cadherin has to occur once again for formation of a secondary neoplasm and establishment of a tumour cell mass (see (1) and (2) above). Therefore, we suggest that the correlation between loss of E-cadherin expression and metastasis in epithelial-derived tumours is a consequence of altered growth factor response which overcomes antiproliferative and proapoptotic signals, rather than an inherent requirement for invasion and motility of the cells. However, the altered growth factor response of cells exhibiting aberrant E-cadherin expression is likely to exacerbate the metastatic phenotype leading to cell invasion and motility, eventually resulting in metastasis of CSCs exhibiting regulation of E-cadherin from the tumour cell mass. Clearly, where expression of E-cadherin at a secondary site is detrimental to CSC establishment, then cells exhibiting irreversible aberrant E-cadherin expression may successfully metastasise.

Whilst we have focused on aberrant E-cadherin expression in the DENT hypothesis, we have not related this effect to the expression of proteins that regulate this process, although these are likely to include the RTK, FGF, and TGF*β* families. Therefore, identification of molecules exhibiting altered expression following aberrant E-cadherin expression within normal epithelium may provide novel targets for further experimental investigation. In addition, the metastatic process, which may involve EMT, is unlikely to be similar to ES cell EMT due to alterations in the underlying genetics of the tumour cells. Therefore, the DENT hypothesis focuses on the effects of aberrant E-cadherin expression in altering growth factor response, rather than inducing an EMT event. Since there is little evidence describing the function of loss of E-cadherin expression alone in epithelial cells or epithelial-derived tumour cells, we believe that analysis of the effects of loss of E-cadherin in the absence of EMT-inducing factors will enhance this field of research.

## 7. Implication of the DENT Hypothesis to Cancer Therapies

The DENT hypothesis reinforces the current view that targeting of CSCs within a tumour cell mass will eliminate tumorigenic and metastatic potential. However, this alone is unlikely to suffice since dedifferentiation of TACs to CSCs could result in establishment of new TSCNs. Therefore, a multiple targeted approach for the elimination of cells within the tumour is likely to be essential. This will require elimination of CSCs and TACs from the tumour, the latter of which may possess the ability to de-differentiate to a CSC phenotype. One possible treatment option for tumour therapy is to induce loss of E-cadherin function in the entire tumour cell mass (via soluble E-cadherin extracellular domain, nAb, or peptide inhibition) to provide a relatively homogenous population of cells where specific inhibition of proliferative pathways associated with the tumorigenic phenotype can be achieved (e.g., FGF signalling). However, such an approach will require the identification of specific pathways within individual tumours, and it is unlikely that all cells within the tumour mass will respond similarly. In addition, successful induction of loss of E-cadherin function in the entire tumour cell population may not be feasible and raises the concern that such treatment could intensify the tumorigenic phenotype. Therefore, a better understanding of signalling pathways which are positively and negatively regulated by E-cadherin expression may permit the development of therapeutics capable of targeting both CSCs and TACs. Currently, there are numerous receptor antagonist and agonist therapeutic agents for the treatment of various malignancies. For example, therapeutics include monoclonal antibodies and small molecules that antagonise factors expressed by tumour cells and the tumour microenvironment. Reagents have been developed to target EGFR and VEGF signalling cascades, which mediate progression of colorectal cancers. In addition, targeting of RTKs using small molecule inhibitors has been utilised to mediate colorectal cancer; both Gefitinib and Erlotinib are reversible EGFR tyrosine kinase inhibitors [110, 111]. Therefore, further elucidation of the signalling pathways within normal epithelium and the tumour microenvironment may allow development of therapeutics to target tumour proliferation on several fronts.

## 8. Conclusions

In summary, we have shown that abrogation of E-cadherin in ES cells results in altered growth factor response, significant changes in the transcriptome and alterations in membrane protein localisation, which correlate with events during tumorigenesis. We have presented the DENT hypothesis to explain events that may occur during neoplasm formation and establishment of a tumour cell mass. The DENT hypothesis presented here exhibits some characteristics of TOFT in that it relies on interactions between the epithelium and stromal cells to induce aberrant E-cadherin expression and subsequent altered growth factor response of epithelial cells. Furthermore, the hypothesis remains faithful to the CSCH and should be viewed as an additional component of this theory that attempts to explain events occurring during the early stages of neoplasia formation. Our aim has been to stimulate discussion of the function of aberrant E-cadherin expression in the early events of tumorigenesis prior to EMT/metastasis and to highlight that loss of E-cadherin during this process may not necessarily reflect a requirement for cell motility and invasion. Rather, we perceive the function of aberrant E-cadherin expression during tumorigenesis to be an integral component of tumour establishment as well as the metastatic spread of tumour cells.

## References

[1] Cavallaro U, Christofori G (2004). Cell adhesion and signalling by cadherins and Ig-CAMs in cancer. *Nature Reviews Cancer*.

[2] Pertz O, Bozic D, Koch AW, Fauser C, Brancaccio A, Engel J (1999). A new crystal structure, Ca2+ dependence and mutational analysis reveal molecular details of E-cadherin homoassociation. *The EMBO Journal*.

[3] Van Roy F, Berx G (2008). The cell-cell adhesion molecule E-cadherin. *Cellular and Molecular Life Sciences*.

[4] Perez-Moreno M, Fuchs E (2006). Catenins: keeping cells from getting their signals crossed. *Developmental Cell*.

[5] McCrea PD, Park J-I (2007). Developmental functions of the P120-catenin sub-family. *Biochimica et Biophysica Acta - Molecular Cell Research*.

[6] Ling K, Bairstow SF, Carbonara C, Turbin DA, Huntsman DG, Anderson RA (2007). Type I*γ* phosphatidylinositol phosphate kinase modulates adherens junction and E-cadherin trafficking via a direct interaction with *μ* 1B adaptin. *Journal of Cell Biology*.

[7] Cavallaro U, Schaffhauser B, Christofori G (2002). Cadherins and the tumour progression: is it all in a switch?. *Cancer Letters*.

[8] Di Croce L, Pelicci PG (2003). Tumour-associated hypermethylation: silencing E-cadherin expression enhances invasion and metastasis. *European Journal of Cancer*.

[9] Margulis A, Zhang W, Alt-Holland A, Crawford HC, Fusenig NE, Garlick JA (2005). E-cadherin suppression accelerates squamous cell carcinoma progression in three-dimensional, human tissue constructs. *Cancer Research*.

[10] Cavallaro U, Christofori G (2004). Multitasking in tumor progression: signaling functions of cell adhesion molecules. *Annals of the New York Academy of Sciences*.

[11] Nieto MA (2002). The snail superfamily of zinc-finger transcription factors. *Nature Reviews Molecular Cell Biology*.

[12] Bolós V, Peinado H, Pérez-Moreno MA, Fraga MF, Esteller M, Cano A (2003). The transcription factor Slug represses E-cadherin expression and induces epithelial to mesenchymal transitions: a comparison with Snail and E47 repressors. *Journal of Cell Science*.

[13] Polette M, Nawrocki-Raby B, Gilles C, Clavel C, Birembaut P (2004). Tumour invasion and matrix metalloproteinases. *Critical Reviews in Oncology/Hematology*.

[14] Nawrocki-Raby B, Gilles C, Polette M (2003). Upregulation of MMPS by soluble E-cadherin in human lung tumor cells. *International Journal of Cancer*.

[15] Crouch S, Spidel CS, Lindsey JS (2004). HGF and ligation of *α*v*β*5 integrin induce a novel, cancer cell-specific gene expression required for cell scattering. *Experimental Cell Research*.

[16] Murai M, Shen X, Huang L (2004). Overexpression of c-met in oral SCC promotes hepatocyte growth factor-induced disruption of cadherin junctions and invasion. *International journal of oncology*.

[17] Khoury H, Naujokas MA, Zuo D (2005). HGF converts ErbB2/Neu epithelial morphogenesis to cell invasion. *Molecular Biology of the Cell*.

[18] Brembeck FH, Rosário M, Birchmeier W (2006). Balancing cell adhesion and Wnt signaling, the key role of *β*-catenin. *Current Opinion in Genetics and Development*.

[19] Watabe M, Nagafuchi A, Tsukita S, Takeichi M (1994). Induction of polarized cell-cell association and retardation of growth by activation of the E-cadherin-catenin adhesion system in a dispersed carcinoma line. *Journal of Cell Biology*.

[20] Doherty P, Walsh FS (1996). CAM-FGF receptor interactions: a model for axonal growth. *Molecular and Cellular Neurosciences*.

[21] Stenzel D, Nye E, Nisancioglu M, Adams RH, Yamaguchi Y, Gerhardt H (2009). Peripheral mural cell recruitment requires cell-autonomous heparan sulfate. *Blood*.

[22] Heasman J, Crawford A, Goldstone K (1994). Overexpression of cadherins and underexpression of *β*-catenin inhibit dorsal mesoderm induction in early xenopus embryos. *Cell*.

[23] Bienz M, Clevers H (2000). Linking colorectal cancer to Wnt signaling. *Cell*.

[24] Gottardi CJ, Gumbiner BM (2004). Distinct molecular forms of *β*-catenin are targeted to adhesive or transcriptional complexes. *Journal of Cell Biology*.

[25] Goodwin M, Kovacs EM, Thoreson MA, Reynolds AB, Yap AS (2003). Minimal mutation of the cytoplasmic tail inhibits the ability of E-cadherin to activate Rac but not phosphatidylinositol 3-kinase. Direct evidence of a role for cadherin-activated Rac signaling in adhesion and contact formation. *Journal of Biological Chemistry*.

[26] Thiery JP (2003). Cell adhesion in development: a complex signaling network. *Current Opinion in Genetics and Development*.

[27] Noren NK, Niessen CM, Gumbiner BM, Burridge K (2001). Cadherin engagement regulates Rho family GTPases. *Journal of Biological Chemistry*.

[28] Martin GS (2003). Cell signaling and cancer. *Cancer Cell*.

[29] Smith AG (2001). Embryo-derived stem cells: of mice and men. *Annual Review of Cell and Developmental Biology*.

[30] Ringe J, Kaps C, Burmester G-R, Sittinger M (2002). Stem cells for regenerative medicine: advances in the engineering of tissues and organs. *Naturwissenschaften*.

[31] Larue L, Antos C, Butz S (1996). A role for cadherins in tissue formation. *Development*.

[32] Lee JM, Dedhar S, Kalluri R, Thompson EW (2006). The epithelial-mesenchymal transition: new insights in signaling, development, and disease. *Journal of Cell Biology*.

[33] Eastham AM, Spencer H, Soncin F (2007). Epithelial-mesenchymal transition events during human embryonic stem cell differentiation. *Cancer Research*.

[34] Spencer HL, Eastham AM, Merry CLR (2007). E-cadherin inhibits cell surface localization of the pro-migratory 5T4 oncofetal antigen in mouse embryonic stem cells. *Molecular Biology of the Cell*.

[35] Ullmann U, In’t Veld P, Gilles C (2007). Epithelial-mesenchymal transition process in human embryonic stem cells cultured in feeder-free conditions. *Molecular Human Reproduction*.

[36] Behr R, Heneweer C, Viebahn C, Denker H-W, Thie M (2005). Epithelial-mesenchymal transition in colonies of rhesus monkey embryonic stem cells: a model for processes involved in gastrulation. *Stem Cells*.

[37] Wicki A, Lehembre F, Wick N, Hantusch B, Kerjaschki D, Christofori G (2006). Tumor invasion in the absence of epithelial-mesenchymal transition: podoplanin-mediated remodeling of the actin cytoskeleton. *Cancer Cell*.

[38] Christiansen JJ, Rajasekaran AK (2006). Reassessing epithelial to mesenchymal transition as a prerequisite for carcinoma invasion and metastasis. *Cancer Research*.

[39] Orsulic S, Kemler R (2000). Expression of Eph receptors and ephrins is differentially regulated by E-cadherin. *Journal of Cell Science*.

[40] Andersen H, Mejlvang J, Mahmood S (2005). Immediate and delayed effects of E-cadherin inhibition on gene regulation and cell motility in human epidermoid carcinoma cells. *Molecular and Cellular Biology*.

[41] Southall PJ, Boxer GM, Bagshawe KD, Hole N, Bromley M, Stern PL (1990). Immunohistological distribution of 5T4 antigen in normal and malignant tissues. *British Journal of Cancer*.

[42] Starzynska T, Wiechowska-Kozlowska A, Marlicz K (1997). The clinical significance of 5T4 antigen in gastric carcinoma. *Gut*.

[43] Starzynska T, Marsh PJ, Schofield PF, Roberts SA, Myers KA, Stern PL (1994). Prognostic significance of 5T4 oncofetal antigen expression in colorectal carcinoma. *British Journal of Cancer*.

[44] Starzynska T, Rahi V, Stern PL (1992). The expression of 5T4 antigen in colorectal and gastric carcinoma. *British Journal of Cancer*.

[45] Starzynska T, Wiechowska-Kozlowska A, Marlicz K (1998). 5T4 oncofetal antigen in gastric carcinoma and its clinical significance. *European Journal of Gastroenterology and Hepatology*.

[46] Carsberg CJ, Myers KA, Stern PL (1996). Metastasis-associated 5T4 antigen disrupts cell-cell contacts and induces cellular motility in epithelial cells. *Molecular Biology of the Cell*.

[47] Lindley LE, Briegel KJ (2010). Molecular characterization of TGF*β*-induced epithelial-mesenchymal transition in normal finite lifespan human mammary epithelial cells. *Biochemical and Biophysical Research Communications*.

[48] Sullivan NJ, Sasser AK, Axel AE (2009). Interleukin-6 induces an epithelial-mesenchymal transition phenotype in human breast cancer cells. *Oncogene*.

[49] Cheng H, Fukushima T, Takahashi N, Tanaka H, Kataoka H (2009). Hepatocyte growth factor activator inhibitor type 1 regulates epithelial to mesenchymal transition through membrane-bound serine proteinases. *Cancer Research*.

[50] Yamauchi Y, Kohyama T, Takizawa H (2010). Tumor necrosis factor-*α* enhances both epithelial-mesenchymal transition and cell contraction induced in A549 human alveolar epithelial cells by transforming growth factor-*β*1. *Experimental Lung Research*.

[51] Smith AG, Heath JK, Donaldson DD (1988). Inhibition of pluripotential embryonic stem cell differentiation by purified polypeptides. *Nature*.

[52] Niwa H, Ogawa K, Shimosato D, Adachi K (2009). A parallel circuit of LIF signalling pathways maintains pluripotency of mouse ES cells. *Nature*.

[53] Paling NRD, Wheadon H, Bone HK, Welham MJ (2004). Regulation of embryonic stem cell self-renewal by phosphoinositide 3-kinase-dependent signaling. *Journal of Biological Chemistry*.

[54] Ying Q-L, Nichols J, Chambers I, Smith A (2003). BMP induction of Id proteins suppresses differentiation and sustains embryonic stem cell self-renewal in collaboration with STAT3. *Cell*.

[55] Ying Q-L, Wray J, Nichols J (2008). The ground state of embryonic stem cell self-renewal. *Nature*.

[56] Soncin F, Mohamet L, Eckardt D (2009). Abrogation of E-cadherin-mediated cell-cell contact in mouse embryonic stem cells results in reversible LIF-independent self-renewal. *Stem Cells*.

[57] Ying Q-L, Wray J, Nichols J (2008). The ground state of embryonic stem cell self-renewal. *Nature*.

[58] Chou Y-F, Chen H-H, Eijpe M (2008). The growth factor environment defines distinct pluripotent ground states in novel blastocyst-derived stem cells. *Cell*.

[59] Arulanandam R, Vultur A, Cao J (2009). Cadherin-cadherin engagement promotes cell survival via Rac1/Cdc42 and signal transducer and activator of transcription-3. *Molecular Cancer Research*.

[60] Massagué J, Chen Y-G (2000). Controlling TGF-*β* signaling. *Genes and Development*.

[61] Vallier L, Mendjan S, Brown S (2009). Activin/Nodal signalling maintains pluripotency by controlling Nanog expression. *Development*.

[62] Vallier L, Alexander M, Pedersen RA (2005). Activin/Nodal and FGF pathways cooperate to maintain pluripotency of human embryonic stem cells. *Journal of Cell Science*.

[63] Tesar PJ, Chenoweth JG, Brook FA (2007). New cell lines from mouse epiblast share defining features with human embryonic stem cells. *Nature*.

[64] Nagaoka M, Koshimizu U, Yuasa S (2006). E-cadherin-coated plates maintain pluripotent ES cells without colony formation. *PLoS ONE*.

[65] Shih I-M, Herlyn M (1993). Role of growth factors and their receptors in the development and progression of melanoma. *Journal of Investigative Dermatology*.

[66] Lázár-Molnár E, Hegyesi H, Tóth S, Falus A (2000). Autocrine and paracrine regulation by cytokines and growth factors in melanoma. *Cytokine*.

[67] Zwick E, Bange J, Ullrich A (2001). Receptor tyrosine kinase signalling as a target for cancer intervention strategies. *Endocrine-Related Cancer*.

[68] Hubbard SR, Miller WT (2007). Receptor tyrosine kinases: mechanisms of activation and signaling. *Current Opinion in Cell Biology*.

[69] Blume-Jensen P, Hunter T (2001). Oncogenic kinase signalling. *Nature*.

[70] Zandi R, Larsen AB, Andersen P, Stockhausen M-T, Poulsen HS (2007). Mechanisms for oncogenic activation of the epidermal growth factor receptor. *Cellular Signalling*.

[71] Plosker GL, Keam SJ (2006). Spotlight on trastuzumab in the management of HER2-positive metastatic and early-stage breast cancer. *BioDrugs*.

[72] Normanno N, Gullick WJ (2006). Epidermal growth factor receptor tyrosine kinase inhibitors and bone metastases: different mechanisms of action for a novel therapeutic application?. *Endocrine-Related Cancer*.

[73] Qian X, Karpova T, Sheppard AM, McNally J, Lowy DR (2004). E-cadherin-mediated adhesion inhibits ligand-dependent activation of diverse receptor tyrosine kinases. *The EMBO Journal*.

[74] Perrais M, Chen X, Perez-Moreno M, Gumbiner BM (2007). E-cadherin homophilic ligation inhibits cell growth and epidermal growth factor receptor signaling independently of other cell interactions. *Molecular Biology of the Cell*.

[75] Andl CD, Rustgi AK (2005). No one-way street: cross-talk between E-cadherin and receptor tyrosine kinase (RTK) signaling: a mechanism to regulate RTK activity. *Cancer Biology and Therapy*.

[76] Jeanes A, Gottardi CJ, Yap AS (2008). Cadherins and cancer: how does cadherin dysfunction promote tumor progression?. *Oncogene*.

[77] Seuntjens E, Umans L, Zwijsen A, Sampaolesi M, Verfaillie CM, Huylebroeck D (2009). Transforming Growth Factor type *β* and Smad family signaling in stem cell function. *Cytokine and Growth Factor Reviews*.

[78] Wikström P, Damber J-E, Bergh A (2001). Role of transforming growth factor-*β*1 in prostate cancer. *Microscopy Research and Technique*.

[79] Teicher BA, Menon K, Alvarez E, Shih PLC, Faul MM (2001). Antiangiogenic and antitumor effects of a protein kinase C*β* inhibitor in human hepatocellular and gastric cancer xenografts. *In Vivo*.

[80] Vrana JA, Stang MT, Grande JP, Getz MJ (1996). Expression of tissue factor in tumor stroma correlates with progression to invasive human breast cancer: paracrine regulation by carcinoma cell- derived members of the transforming growth factor *β* family. *Cancer Research*.

[81] Lee C, Sintich SM, Mathews EP (1999). Transforming growth factor-*β* in benign and malignant prostate. *Prostate*.

[82] Massagué J, Wotton D (2000). Transcriptional control by the TGF-*β*/Smad signaling system. *The EMBO Journal*.

[83] Risbridger GP, Schmitt JF, Robertson DM (2001). Activins and inhibins in endocrine and other tumors. *Endocrine Reviews*.

[84] Coerver KA, Woodruff TK, Finegold MJ, Mather J, Bradley A, Matzuk MM (1996). Activin signaling through activin receptor type II causes the cachexia-like symptoms in inhibin-deficient mice. *Molecular Endocrinology*.

[85] Halaban R, Kwon BS, Ghosh S, Delli Bovi P, Baird A (1988). bFGF as an autocrine growth factor for human melanomas. *Oncogene Research*.

[86] Wang Y, Becker D (1997). Antisense targeting of basic fibroblast growth factor and fibroblast growth factor receptor-1 in human melanomas blocks intratumoral angiogenesis and tumor growth. *Nature Medicine*.

[87] Huang A, Wright JA (1994). Fibroblast growth factor mediated alterations in drug resistance, and evidence of gene amplification. *Oncogene*.

[88] Skarda J, Fridman E, Plevova P (2006). Prognostic value of hMLH1 and hMSH2 immunohistochemical expression in non-small cell lung cancer. A tissue microarray study. *Biomedical papers of the Medical Faculty of the University Palacký, Olomouc, Czechoslovakia*.

[89] Miyake A, Konishi M, Martin FH (1998). Structure and expression of a novel member, FGF-16, of the fibroblast growth factor family. *Biochemical and Biophysical Research Communications*.

[90] Ruotsalainen T, Joensuu H, Mattson K, Salven P (2002). High pretreatment serum concentration of basic fibroblast growth factor is a predictor of poor prognosis in small cell lung cancer. *Cancer Epidemiology Biomarkers and Prevention*.

[91] Granato AM, Nanni O, Falcini F (2004). Basic fibroblast growth factor and vascular endothelial growth factor serum levels in breast cancer patients and healthy women: useful as diagnostic tools?. *Breast Cancer Research*.

[92] Giri D, Ropiquet F, Ittmann M (1999). Alterations in expression of basic fibroblast growth factor (FGF) 2 and its receptor FGFR-1 in human prostate cancer. *Clinical Cancer Research*.

[93] Amit M, Carpenter MK, Inokuma MS (2000). Clonally derived human embryonic stem cell lines maintain pluripotency and proliferative potential for prolonged periods of culture. *Developmental Biology*.

[94] Allerstorfer S, Sonvilla G, Fischer H (2008). FGF5 as an oncogenic factor in human glioblastoma multiforme: autocrine and paracrine activities. *Oncogene*.

[95] Curtis HJ (1965). Formal discussion of: somatic mutations and carcinogenesis. *Cancer Research*.

[96] Loeb LA (2001). A mutator phenotype in cancer. *Cancer Research*.

[97] Mintz B, Illmensee K (1975). Normal genetically mosaic mice produced from malignant teratocarcinoma cells. *Proceedings of the National Academy of Sciences of the United States of America*.

[98] Rossant J, Papaioannou VE (1985). Outgrowth of embryonal carcinoma cells from injected blastocysts in vitro correlates with abnormal chimera development in vivo. *Experimental Cell Research*.

[99] Sonnenschein C, Soto AM (2008). Theories of carcinogenesis: an emerging perspective. *Seminars in Cancer Biology*.

[100] Fey C (1950). Virchow is opposition to the therapeutic nihilismus of his times. *Hippokrates*.

[101] Winter K (1952). Significance of young Rudolf Virchow in present day thinking. *Zeitschrift für ärztliche Fortbildung*.

[102] Gruber GB (1952). When Virchow was a young physician. *Virchows Archiv*.

[103] WAIL SS (1950). Mechanical and anti-evolutionary conceptions of Virchow's cellularity and progress of pathology in the Soviet Union. *Sovetskaia Meditsina*.

[104] Lapidot T, Sirard C, Vormoor J (1994). A cell initiating human acute myeloid leukaemia after transplantation into SCID mice. *Nature*.

[105] Bonnet D, Dick JE (1997). Human acute myeloid leukemia is organized as a hierarchy that originates from a primitive hematopoietic cell. *Nature Medicine*.

[106] Al-Hajj M, Wicha MS, Benito-Hernandez A, Morrison SJ, Clarke MF (2003). Prospective identification of tumorigenic breast cancer cells. *Proceedings of the National Academy of Sciences of the United States of America*.

[107] Zhang P, Zuo H, Ozaki T, Nakagomi N, Kakudo K (2006). Cancer stem cell hypothesis in thyroid cancer. *Pathology International*.

[108] Odoux C, Fohrer H, Hoppo T (2008). A stochastic model for cancer stem cell origin in metastatic colon cancer. *Cancer Research*.

[109] Clarke MF, Dick JE, Dirks PB (2006). Cancer stem cells—perspectives on current status and future directions: AACR workshop on cancer stem cells. *Cancer Research*.

[110] Kelly PN, Dakic A, Adams JM, Nutt SL, Strasser A (2007). Tumor growth need not be driven by rare cancer stem cells. *Science*.

[111] Yoo M-H, Hatfield DL (2008). The cancer stem Cell theory: is it correct?. *Molecules and Cells*.

[112] Mani SA, Guo W, Liao M-J (2008). The epithelial-mesenchymal transition generates cells with properties of stem cells. *Cell*.

[113] Hermiston ML, Wong MH, Gordon JI (1996). Forced expression of E-cadherin in the mouse intestinal epithelium slows cell migration and provides evidence for nonautonomous regulation of cell fate in a self-renewing system. *Genes and Development*.

[114] Libusova L, Stemmler MP, Hierholzer A, Schwarz H, Kemler R (2010). N-cadherin can structurally substitute for E-cadherin during intestinal development but leads to polyp formation. *Development*.

[115] Conti L, Pollard SM, Gorba T (2005). Niche-independent symmetrical self-renewal of a mammalian tissue stem cell. *PLoS Biology*.

[116] Stingl J, Eirew P, Ricketson I (2006). Purification and unique properties of mammary epithelial stem cells. *Nature*.

[117] Bryden AAG, Freemont AJ, Clarke NW, George NJR (1999). Paradoxical expression of E-cadherin in prostatic bone metastases. *BJU International*.

[118] Bukholm IK, Nesland JM, Børresen-Dale A-L (2000). Re-expression of E-cadherin, *α*-catenin and *β*-catenin, but not of *γ*- catenin, in metastatic tissue from breast cancer patients. *Journal of Pathology*.

[119] Gaspar RH, De los Toyos JR, Marcos CA, Riera JR, Sampedro A (1999). Quantitative immunohistochemical analyses of the expression of E- cadherin, thrombomodulin, CD44H and CD44v6 in primary tumours of pharynx/larynx squamous cell carcinoma and their lymph node metastases. *Analytical Cellular Pathology*.

